# Editorial: Unlocking the potential of the microbiome in cancer therapy

**DOI:** 10.3389/fmicb.2026.1950129

**Published:** 2026-09-04

**Authors:** Roshan Kumar, Babak Pakbin, Mona Roshan Singh, Bharathi Muruganantham

**Affiliations:** 1 Department of Obstetrics and Gynecology, Division of Gynecologic Oncology, Medical College of Wisconsin, Milwaukee, WI, United States; 2 Michels Center for Cancer Discovery, Medical College of Wisconsin, Milwaukee, WI, United States; 3 Post-Graduate Department of Zoology, Magadh University, Bodh Gaya, Bihar, India; 4 Department of Food Science and Technology, Texas A&M University, College Station, TX, United States; 5 Centre for Bioinformatics, Karpagam Academy of Higher Education, Coimbatore, Tamil Nadu, India; 6 Department of Biochemistry, Karpagam Academy of Higher Education, Coimbatore, Tamil Nadu, India

**Keywords:** cancer, interaction, metabolome, microbiome, microenvironment

Cancer remains a formidable global challenge, shaped by genetic, environmental, and lifestyle factors. The advent of high-throughput sequencing has deepened our understanding of these relationships, revealing the microbiome as a key player in shaping the tumor microenvironment, host immune tone, treatment response, and clinical outcome (Nejman et al., 2020; Battaglia et al., 2024; Kumar et al., 2025). Microbial communities produce metabolites, regulate inflammation, alter mucosal integrity, metabolize dietary and pharmacological compounds, and influence whether a patient tolerates or responds to chemotherapy, radiotherapy, immunotherapy, or surgery. High discrepancies in cancer incidence, progression, and outcomes across populations further highlight the importance of integrating microbial, biological, and socioeconomic factors into cancer research.

The idea of this research topic, “*Unlocking the Potential of the Microbiome in Cancer Therapy*,” was to elucidate the multifaceted roles of the microbiome in cancer therapy. Our goal was to deepen the understanding of how microbiome variations influence individual responses to cancer treatments and to investigate how microbial communities can be intentionally altered to enhance therapeutic outcomes. We were particularly interested in how microbial metabolites and immune system interactions can be harnessed to develop novel, more effective therapies. The Research Topic invited contributions spanning probiotic and postbiotic interventions, targeted antimicrobial strategies, computational models integrating host genetics with microbiome data, microbiome-mediated immune modulation, and microbial metabolites as biomarkers. The resulting 24 publications span original research, reviews, and systematic synthesis across Frontiers in Microbiology and participating journals.

Through this Research Topic, we wanted to clarify how distinct cancers harbor distinct microbial signatures across anatomically and clinically relevant niches. In pancreatic cancer, Kim et al. used multi-site 16S profiling of saliva, feces, and blood from patients and controls to show enrichment of *Lactobacillus* in saliva, *Enterobacter* in feces, and *Prevotella* in blood, alongside reduced microbial network interactions in cancer patients to emphasize that cancer-associated dysbiosis may be distributed across body sites rather than confined to the tumor or intestine alone. This multi-compartment view is extended by work in colorectal cancer (CRC), where noninvasive detection remains a central clinical goal. Wu Z. et al. compared fecal and salivary signatures across metastatic CRC, non-metastatic CRC, and healthy controls, finding that fecal ASV classifiers outperformed salivary classifiers for CRC detection. Complementing this diagnostic perspective, Chen, Guan et al. profiled the polyp-adenoma-carcinoma sequence by metagenomic sequencing and reported a random forest model capable of distinguishing intramucosal carcinoma, accompanied by metabolic reprogramming toward a pro-tumorigenic phenotype.

The hepatobiliary tract emerges as another key setting in which microbiome-metabolome interactions may inform risk and prognosis. Wang et al. integrated bile 16S sequencing and LC-MS metabolomics in cholangiocarcinoma and choledocholithiasis, revealing altered biliary microbial compositions and microbial-metabolite interactions associated with malignancy. In hepatocellular carcinoma (HCC), Chen, Wang et al. reported that integrated microbiome-metabolome profiling could predict early recurrence, with a 20-genus microbial signature achieving an AUC of 0.81 and a 20-metabolite panel achieving an AUC of 0.958. Ramadan et al. added a clinically specific lens by studying diabetic patients with HCC after hepatitis C virus eradication, identifying reduced diversity, enrichment of *Treponema_2* and *Klebsiella*, and depletion of *Faecalibacterium*. Altogether, these studies suggest that the gut-liver-bile ecosystem may encode clinically actionable information about inflammation, metabolic status, and recurrence risk.

Microbial profiling within and around tumors further highlights the importance of local ecology. In a 4NQO murine model of head and neck squamous cell carcinoma, Xie et al. found reduced intratumoral alpha diversity, enrichment of *Aggregatibacter* and *Pseudomonas*, and depletion of *Lactobacillus*. In lung adenocarcinoma, Yan et al. paired tumor core and airway lumen bronchoalveolar lavage fluid microbiomes from 77 patients, identifying *Bacillus* enrichment in tumors and associations with invasiveness and an immunosuppressive microenvironment marked by upregulated *VSIR, TIGIT*, and *PD*-*L1*. Within CRC tissues, Ma et al. compared tumor, paracancerous, and fecal samples, showing enrichment of *Fusobacterium* and *Escherichia*–*Shigella* in tumors and a correlation between *E. coli* and tumor size. These studies converge on a central principle that cancer microbiomes are spatially structured, and their clinical meaning depends on where they are measured.

Beyond description, the field now requires mechanistic clarity. One of the strongest contributions of this Research Topic is its emphasis on pathways connecting microbes to tumor biology. Chen X. et al. examined spatial microbiome-metabolic crosstalk in CRC and reported that depletion of *Streptococcus* and *Acetivibrio* disrupts *butyrate-HDAC sign*aling, leading to PDCD1 hyperacetylation and CD8+ T-cell exhaustion. This work links microbial spatial organization to immune dysfunction and proposes a novel consensus molecular subtype biomarker. Mechanistic synthesis is also provided by Bi et al., who revisited berberine for prevention and treatment of *Fusobacterium nucleatum*-induced CRC, emphasizing stage-specific anti-CRC effects mediated through E-cadherin, Wnt/β-catenin, JAK/STAT, and MAPK/ERK pathways.

The same mechanistic logic points toward new microbial therapeutics. Kabwe et al. showed that *bacteriophage* FNU1 can negate *F. nucleatum*-induced cell growth, migration, and chemotherapy resistance in gastrointestinal cancer cells, illustrating the possibility of precision microbial editing rather than broad antimicrobial depletion. Liu J. et al. identified *Bacteroides thetaiotaomicron* M60 metalloproteinases as mucolytic agents capable of degrading *pseudomyxoma peritonei* mucus, opening a complementary strategy in which bacterial enzymes are repurposed against the physical barriers of mucinous cancers. These studies expand microbiome oncology beyond biomarkers, toward phages, enzymes, metabolites, and pathway-directed interventions.

A third objective of this Research Topic is to evaluate how microbiome modulation may improve cancer treatment outcomes. Perioperative care is one of the most mature areas for translation. In an umbrella review of 11 meta-analyses, Gao S. et al. reported that probiotics and synbiotics significantly reduced postoperative complications after CRC resection, including overall infections (OR 0.49), surgical site infection (OR 0.58), urinary tract infection (OR 0.39), pneumonia (OR 0.34), and diarrhea (OR 0.41). Dietary modulation offers another practical route. McCartney et al. reviewed short-term preoperative dietary fiber interventions in CRC treatment, emphasizing their capacity to modify gut microbiota and potential to improve surgical and oncological outcomes. These papers frame the microbiome as a modifiable perioperative variable, not merely a correlate of disease.

Radiotherapy represents a second arena in which the microbiome may influence both efficacy and toxicity. Gao Y. et al. synthesized evidence that fecal microbiota metabolites shape radiotherapy outcomes through signaling pathways and the tumor immune microenvironment. Wu B. et al. further reviewed how gut and oral microbiota, including short-chain fatty acids, regulate radiosensitivity and radiotolerance through DNA damage repair, immune responses, and metabolic reprogramming. These reviews highlight an important therapeutic tension: the same microbial or metabolic axis may enhance tumor radiosensitivity while protecting normal tissue, or conversely promote resistance, depending on host, tumor, and treatment context. In postoperative rectal cancer, Chen H. et al., compared 40 patients receiving stent-based diversion technique or prophylactic double-lumen ileostomy and reported that stent-based diversion better preserved gut microbiota diversity, promoted restoration of beneficial bacteria, and was associated with a lower anastomotic stricture rate (4.76% vs. 31.58%). This contribution reinforces the idea that surgical strategy itself can be microbiome-modifying.

The Research Topic also includes reviews that organize the rapidly expanding literature into translational frameworks. Bai et al. synthesized gut microbiota and CRC through mechanistic insights, diagnostic advances, and microbiome-based therapeutic strategies, proposing a multi-omics minimal diagnostic signature and a pathway-therapy-microbiome matching framework for precision oncology. Zheng et al. reviewed the urinary microbiome in urological cancers, emphasizing dysbiosis in bladder and prostate cancers and mechanisms involving inflammation and immunomodulation. Li and Liu developed a microbiota-immuno-metabolic axis framework for liver cell carcinoma, connecting gut dysbiosis, barrier impairment, and metabolites to hepatocarcinogenesis and treatment response. Yuan et al. focused on the gut–pancreatic axis, describing how gut microbiota may migrate to the pancreas and reshape the immunosuppressive tumor microenvironment of pancreatic ductal adenocarcinoma, with implications for microbiome-targeted immunotherapy.

Clinical translation will also require careful attention to intervention safety, standardization, and special patient populations. Liu X. et al. reviewed fecal microbiota transplantation as an adjuvant strategy for bladder cancer through the gut-bladder axis, while emphasizing unresolved challenges in donor selection, manufacturing, regulation, and safety. Yao et al. broadened the scope to critically ill patients with septic shock, comparing those with and without malignancies and reporting that cancer patients had a lower pathogen detection rate, shorter ICU stays, distinct resistome features, and *IL-6* as a prognostic indicator. This contribution reminds the field that cancer microbiome science must contend with antimicrobial exposure, immune suppression, acute illness, and resistance ecology, all of which can alter both microbial readouts and therapeutic risk.

These contributions show a field moving from descriptive microbiome catalogs toward mechanism, prediction, and intervention. The microbiome actively influences carcinogenesis, immune state, treatment response, toxicity, surgical recovery, and recurrence. Near-term opportunities include non-invasive diagnostics, perioperative probiotics, metabolite-guided radiotherapy, and microbial biomarkers for recurrence, alongside more experimental strategies such as phage therapy, microbial enzymes, FMT, and emerging approaches such as engineered bacteria. Many associations require causal validation in longitudinal cohorts and multi-center trials. Sampling methods, contamination control, and bioinformatic pipelines need standardization to allow comparison across studies. Most importantly, microbiome-based therapy must account for inter-individual variability rather than assume a universal healthy microbiome. Future work should integrate metagenomics, metatranscriptomics, metabolomics, spatial profiling, tumor genomics, diet, and clinical outcomes. By linking ecological insight to clinical action, this Research Topic provides a foundation for a future in which the microbiome is used not only to understand cancer, but to improve cancer therapy.

## References

[B1] Battaglia T. W. Mimpen I. L. Traets J. J. H. van Hoeck A. Zeverijn L. J. Geurts B. S. . (2024). A pan-cancer analysis of the microbiome in metastatic cancer. Cell 187, 2324–2335.e19. doi: 10.1016/j.cell.2024.03.021 38599211

[B2] Kumar R. Duyar-Ayerdi S. Sundaresan A. Srinivasasainagendra V. Pedamallu C. S. Behring M. . (2025). Race-related host and microbe transcriptomic signatures in triple-negative breast cancer. NPJ Breast Cancer 11:87. doi: 10.1038/s41523-025-00806-y PMC12334597 40781241

[B3] Nejman D. Livyatan I. Fuks G. Gavert N. Zwang Y. Geller L. T. . (2020). The human tumor microbiome is composed of tumor type-specific intracellular bacteria. Science 368, 973–980. doi: 10.1126/science.aay9189 PMC7757858 32467386

